# Successful cardiopulmonary resuscitation following minimally invasive pectus excavatum repair: A case report

**DOI:** 10.1016/j.ijscr.2019.10.055

**Published:** 2019-10-31

**Authors:** Kyle J. Glithero, John J. Tackett, Kenneth DeMason, Cathy A. Burnweit

**Affiliations:** a Department of Pediatric Surgery, Nicklaus Children’s Hospital, Medical Arts Building, 3200 SW 60th Court, Suite 201, Miami, FL 33155, USA; b University of Florida, Gainesville, FL 32611-8105, USA

**Keywords:** Pectus excavatum, Minimally invasive pectus excavatum repair, Nuss procedure, Cardiopulmonary resuscitation, Cardiac arrest, Case report

## Abstract

**Introduction:**

Pectus excavatum is the most common congenital chest wall deformity. The minimally invasive repair of pectus excavatum (MIRPE) is the most commonly practiced method of surgical treatment and there is concern that the pectus bar will prevent effective CPR. There are no recorded cases of successful cardiopulmonary resuscitation with pectus bars in place.

**Presentation of case:**

A 17-year-old male with pectus excavatum underwent MIRPE. Two years later, he experienced out-of-hospital cardiac arrest and underwent successful cardiopulmonary resuscitation (CPR) in the field with a pectus bar in place.

**Discussion:**

Successful CPR is possible after MIRPE. Clear identification of patients who have undergone MIRPE and education of CPR providers in providing effective chest compressions and defibrillation for this patient population is necessary.

**Conclusion:**

This is the first documented case of successful CPR in a patient with a pectus bar in place who experienced out-of-hospital cardiac arrest.

## Highlights

• Pectus excavatum is the most common chest wall deformity.
• Correction involves placement of a steel pectus bar(s) in a substernal position.
• Historically, there has been concern that a pectus bar prevents effective CPR.
• Successful CPR with a pectus bar in place was achieved in the field.

## 1 Introduction

Pectus excavatum is the most common congenital chest wall deformity, accounting for 90% of chest wall deformities and occurring at a 4:1 male to female ratio [1,2]. The minimally invasive repair of pectus excavatum (MIRPE) is the most commonly practiced method of surgical correction [3]. During a MIRPE 1or 2 stainless steel pectus bars are placed across the chest in the substernal position in order to correct the defect. An association between pectus excavatum and cardiac abnormalities is also well established [4]. Due to the relative prevalence of pectus excavatum, its association with cardiac anomalies, and the popularity of the MIRPE, there has been interest in the efficacy of cardiopulmonary resuscitation (CPR) in this population. Until now, successful CPR in a patient with a pectus bar has not been described [5,6]. We present the case of a 17-year-old male who underwent MIRPE at tertiary care children’s hospital, and prior to bar removal, experienced cardiac arrest with documented ventricular fibrillation followed by return of spontaneous circulation after successful CPR. This case is reported in congruence with Surgical Case Report (SCARE) Guidelines [7].

## 2 Presentation of case

**Fig. 1:**
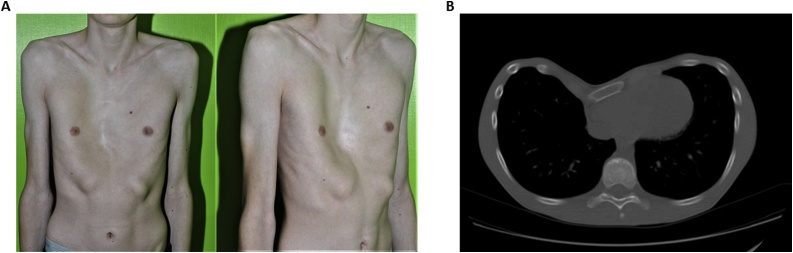
A, Preoperative photograph of chest showing pectus defect. B, Preoperative computerized tomography of the chest showing pectus excavatum deformity.

A 17-year-old tall, thin male presented with pectus excavatum. His body mass index was 15.3 kg/m^2^ (height 179 cm, weight 50 kg). On physical exam, he had a regular cardiac rate and rhythm, soft systolic click at the lower left sternal border, and soft vibratory systolic murmur at the left midsternal border. His chest defect showed a greater depression on the right (Fig. 1A). Although he demonstrated phenotypic traits of Marfan syndrome, genetic testing was negative. Preoperative electrocardiogram (ECG) showed right axis deviation, incomplete right bundle branch block, first degree atrioventricular block and a normal QT interval. The preoperative echocardiogram showed mild mitral and tricuspid valve prolapse with mild tricuspid regurgitation and normal ventricular function and a normal aortic root and ascending aorta. Pulmonary function tests (PFTs) were consistent with a mild restrictive pattern. Computerized tomography (CT) of the chest demonstrated central depression of the sternum with mass effect on the right cardiac chambers (Fig. 1B). Haller and correction indexes were calculated at 4.3 and 39%, respectively.

**Fig. 2:**
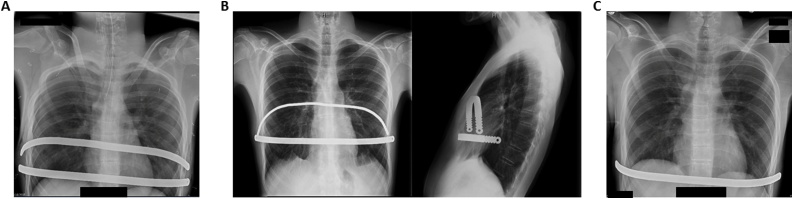
A, Postoperative anteroposterior chest radiograph showing two pectus bars in place. B, Posteroranterior and lateral chest radiographs showing bar slippage. C, Anteroposterior chest radiograph after removal of slipped bar.

In mid-2015, the patient underwent an uncomplicated MIRPE with two 13-inch pectus bars secured to the ribs with FiberWire®. The postoperative chest x-ray was significant only for tiny bilateral apical pneumothoraces secondary to the positive pressure used during thoracoscopy (Fig. 2A). The patient was discharged home on postoperative day four in good condition. One month postoperatively, he was found to have protrusion of a FiberWire® suture securing the right upper bar, necessitating FiberWire® removal under local anesthesia. After removal of the FiberWire®, there was some slippage of the superior bar and it was removed six months after the pectus repair (Fig. 2B, C). The lower bar was left untouched. At two weeks after the superior bar removal the patient was healing well with no change in the correction of his pectus defect and he was cleared for all activities.

**Fig. 3:**
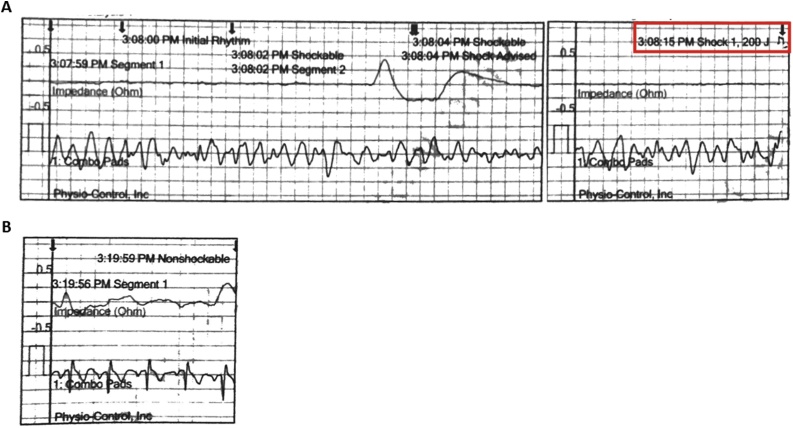
A, Rhythm strip from AED showing ventricular fibrillation and deliverance of 200 J shock (outlined in red). B, Rhythm strip showing normal sinus rhythm after cessation of chest compressions and palpable pulse.

Twenty-three months after pectus repair, the patient collapsed while at a beachfront state park with his Eagle Scout group. The event was witnessed by the scout leader who noted that he fell backwards on to the pavement, hit his head, and then had jerking movements of his arms. He was unresponsive, not breathing, and had turned grey in color. She began chest compressions. Two lifeguards came to assist and noted that the boy was “purple”. One lifeguard took over compressions from the scout leader. The scout leader called the patient’s mother who referred her to the patient’s medical alert necklace stating that the patient had a substernal steel bar and that extra force was required during CPR. An automatic external defibrillator (AED) was obtained, applied, and turned on. It was estimated that approximately 7 min. had passed since compressions had started. At 15:07:59 the AED demonstrated ventricular fibrillation (Fig. 3A). A shock was advised, delivered at 15:08:15, and compressions were continued. No further shocks were administered. The Fire Rescue team arrived at 15:19:00 and return of spontaneous circulation was documented at 15:19:59 (Fig. 3B). Vitals at 15:23:40 showed heart rate of 108 and normal blood pressure and oxygen saturation. The patient remained unresponsive, with upward eye deviation. A 12-lead ECG showed sinus rhythm. The patient was stabilized and evacuated via helicopter to the nearest trauma center.

Upon arrival to the hospital, advance trauma life support was initiated. An endotracheal tube was placed to protect the patient’s airway. There was no evidence of injury on primary or secondary survey. Laboratory examinations were significant for elevated white blood cell count and an increased anion gap with mixed metabolic/respiratory acidosis. Troponin-I was elevated at 0.350 ng/mL, but subsequently normalized. Creatine phosphokinase peaked at 2346 unit/L. Imaging, including CT and CT angiogram of the head and cervical spine, was normal. The patient was admitted to the intensive care unit. A lumbar puncture did not show evidence of an infection. Cultures from the endotracheal tube grew Pseudomonas oryzihabitans. Magnetic resonance imaging of the brain showed no acute intracranial abnormality. An electroencephalogram (EEG) showed mild bilateral slowing. An echocardiogram showed an ejection fraction of 55% and trace tricuspid regurgitation. The patient was subsequently extubated and regained baseline functioning with no focal neurological deficits. He was discharged home on hospital day five with levofloxacin and levetiracetam. After discharge a 48-h EEG study demonstrated epileptiform activity over bi-frontal regions consistent with an area of cortical hyper-excitability from which seizures may arise.

**Fig. 4:**
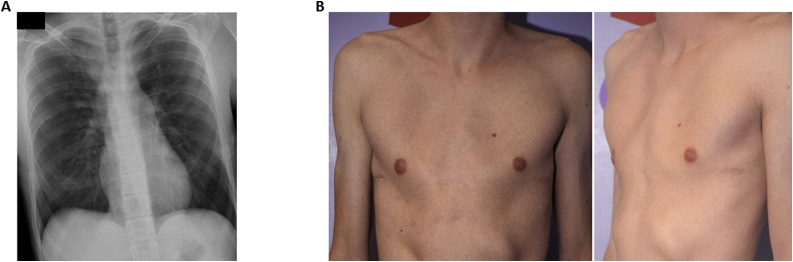
A, Anteroposterior chest radiograph after planned removal of final bar. B, Postoperative photograph of chest.

The patient continues on anti-seizure medication and has not experienced subsequent seizures. He has returned to university to complete his studies in math and biochemistry where he has earned a 4.0 grade point average and is ranked among the top in his class. His second bar was removed without incident as scheduled during the summer of 2019 with good correction of his pectus defect (Fig. 4A, B).

## 3 Discussion

The 2010 guidelines on CPR from the American Heart Association (AHA) emphasize the importance of optimizing CPR quality for increased success rate of shock delivery and survival [8]. To this end, the ABCs (Airway, Breathing, Chest compressions) of basic life support were changed to C-A-B, placing an emphasis on effective chest compressions to a depth of at least 2 in. with minimal time between delivering a shock and resuming compressions. To achieve this goal, certain adjustments may have to be implemented when performing CPR after placement of a pectus bar.

It has been suggested that in this patient population, chest compression may require a greater force to be effective and the defibrillator pads should be placed front to back in order to reduce dissipation of electrical current by the pectus bar [9]. For these reasons, it is important that every patient who undergoes a MIRPE wear a medical necklace/bracelet alerting health care providers to the presence of the pectus bar and the need to adjust CPR accordingly. Due to the relatively high incidence of pectus excavatum and the widespread implementation of MIRPE for correction of pectus excavatum, the performance of CPR in patients with pectus excavatum and/or MIRPE may deserve attention in the next iteration of the AHA guidelines on CPR.

Despite association of pectus excavatum with cardiac abnormalities, the incidence of sudden cardiac arrest after MIRPE appears low [3,10]. Upon review of the literature just two case reports were found that described sudden cardiac arrest outside of the hospital after MIRPE [5,6]. Both patients received CPR and attempted defibrillation, however, both patients died (Table 1), again underlying the need for proper education of CPR providers caring for patients after MIRPE.

**Table 1:** Reported cases of out-of-hospital cardiac arrest with sternal bar in place after MIRPE.

Year of publication	Journal	Author	Patient age (years)	Sex	Cardiac rhythm	Underlying pathology	Time from MIRPE to arrest (months)	Outcome
2005	J. Pediatr. Surgery	Garret K, et al.	21	M	Vfib	Mitral valve prolapse	36	Death
2015	Acute Med & Surgery	Nakahara O, et al.	14	M	Asystole	Right ventricular hypertrophy	15	Death
	This case	Glithero KJ, et al.	17	M	Vfib	Seizure disorder	23	Survival

MIRPE, minimally invasive repair of pectus excavatum; M, male; Vfib, ventricular fibrillation.

It has also been hypothesized that lower placement of the bar may improve effectiveness of chest compressions [5]. Of note, this patient originally had two bars placed, but the superior bar had been removed due to slippage prior to the cardiac arrest. The lower position of the remaining bar possibly contributed to successful resuscitation.

## 4 Conclusion

This is the first published description of successful CPR after sudden cardiac arrest with a pectus bar in place. Although this is a rare phenomenon, it should be standard of care that all patients who undergo MIRPE wear a medical bracelet/necklace that alerts CPR providers to the presence of the pectus bar and that adjustments may be needed for the effective delivery of CPR. Due to the relatively high incidence of pectus excavatum and popularity of MIRPE, perhaps this chest wall deformity should be addressed in the next iteration of the AHA guidelines on CPR.

## Sources of funding

None.

## Ethical approval

A single retrospective case study that reports the observation of a single subject receiving the normal standard of care (no new or novel procedures) is generally not considered research and thus is exempt from needing IRB approval.

## Consent

Written informed consent was obtained from the patient for publication of this case report and accompanying images. A copy of the written consent is available for review by the Editor-in-Chief of this journal on request.

## Author’s contribution

Kyle Glithero: Investigation, Data Curation, Writing – Original Draft, Writing – Review & Editing, Visualization. John Tackett: Writing – Review & Editing. Kenneth DeMason: Data Curation, Review & Editing. Cathy Ann Burnweit: Conceptualization, Investigation, Resources, Writing – Review & Editing, Supervision.

## Registration of research studies

Not applicable. As per the Research Registry, “We do not register case reports that are not first-in-man or animal studies.”

## Guarantor

Cathy Burnweit and Kyle Glithero.

## Provenance and peer review

Not commissioned, externally peer-reviewed.

## Declaration of Competing Interest

None.
